# Supplementary material for: Screening the Presence of Non-Typhoidal Salmonella in Different Animal Systems and the Assessment of Antimicrobial Resistance
Source: Animals (Basel). 2021 May 24;11(6):1532. doi: 10.3390/ani11061532 (PMC8225015; doi:10.3390/ani11061532)
Supplement: Supplementary file 1 [file animals-11-01532-s001.zip › Fig S1 .pdf]

Supplemental Figure 1. Predicted AMR prevalence from Figure 3 by system and sampling types

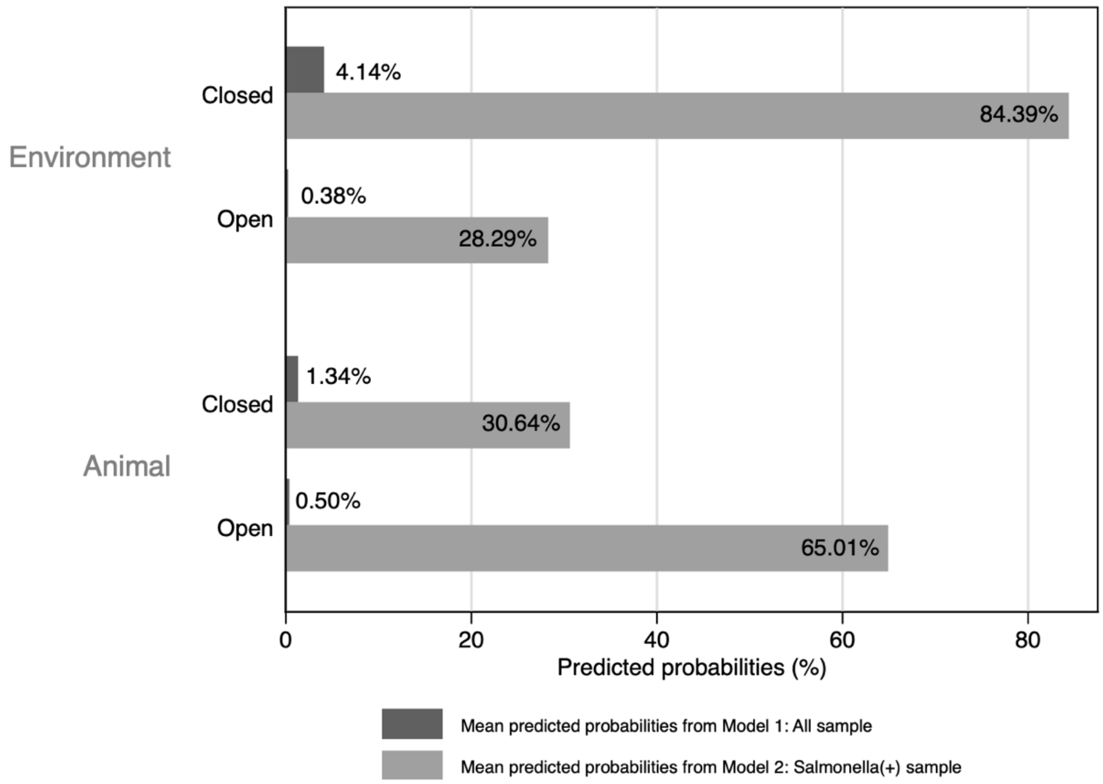

Notes: Mean of the predicted AMR prevalence from Model 1 and 2 (Figure 3). Y-axis classifies our sample into system and sampling types.
