# Supplementary material for: Screening the Presence of Non-Typhoidal Salmonella in Different Animal Systems and the Assessment of Antimicrobial Resistance
Source: Animals (Basel). 2021 May 24;11(6):1532. doi: 10.3390/ani11061532 (PMC8225015; doi:10.3390/ani11061532)
Supplement: Supplementary file 1 [file animals-11-01532-s001.zip › Table S2.pdf]

Table S2. Results of the multivariate logistic regression model: exploratory analysis

Prevalence of AMR Salmonella	Model 3				Model 4			
Variables	OR	OR 95%CI	SE	Pr(>|z|)	OR	OR 95%CI	SE	Pr(>|z|)
**System type**[a] (ref.= free-range)								
Closed	3.90**	1.36,11.20	2.10	0.011	1.42	0.45,4.48	0.83	0.381
**Sampling type**[a] (ref.= animal)								
environment	0.89	0.28,2.84	0.53	0.847	1.97	0.64,5.99	1.12	0.137
**Serogroup type** (ref.= serogroup B)								
Serogroup C1	0.18**	0.04,0.74	0.13	0.018	0.28**	0.07,1.04	0.19	0.050
Serogroup C2-C3	1.09	0.13,9.37	1.20	0.931	1.02	0.09,12.08	1.28	0.993
Serogroup D	0.05**	0.00,0.55	0.06	0.015	0.07**	0.01,0.71	0.09	0.024
Serogroup E1	1.07	0.05,20.81	1.62	0.965	1.20	0.06,25.27	1.87	0.906
Serogroup N/D	0.00***	0.00,0.00	0.00	<0.001	0.32	0.07,1.34	0.24	0.121
Constant term	0.94	0.32,2.71	0.51	0.903	0.83	0.24,2.85	0.52	0.771
Number of observations	3,851				98			
Pseudo R^2	65.70				16.79			
AIC	191.11				130.55			
VCE[c]	Robust				Robust			
Sample	All samples collected				*Salmonella* (+) isolates			

Reference group (ref.) stands for backyard chicken farms and serogroup B. Our dependent variable indicates the presence of resistance to at least one antimicrobial for *Salmonella*. SE stands for standard error, whereas OR is for Odds Ratios. [a]Some categories were dropped as they did not present variation over AMR prevalence (i.e., industrial dairy farm and other backyard animals). [b]Category dropped in Model 2 due to lack of variation over AMR prevalence. [c]Robust standard errors were estimated.: * p<0.1, ** p<0.05, *** p<0.01
